# Amphibian Segmentation Clock Models Suggest How Large Genome and Cell Sizes Slow Developmental Rate

**DOI:** 10.1093/iob/obae021

**Published:** 2024-06-19

**Authors:** A Taylor, A Prasad, R Lockridge Mueller

**Affiliations:** Department of Biology, Colorado State University, Fort Collins, CO 80523, USA; Department of Chemical and Biological Engineering, Colorado State University, Fort Collins, CO 80523, USA; Department of Biology, Colorado State University, Fort Collins, CO 80523, USA

## Abstract

Evolutionary increases in genome size, cell volume, and nuclear volume have been observed across the tree of life, with positive correlations documented between all three traits. Developmental tempo slows as genomes, nuclei, and cells increase in size, yet the driving mechanisms are poorly understood. To bridge this gap, we use a mathematical model of the somitogenesis clock to link slowed developmental tempo with changes in intra-cellular gene expression kinetics induced by increasing genome size and nuclear volume. We adapt a well-known somitogenesis clock model to two model amphibian species that vary 10-fold in genome size: *Xenopus laevis* (3.1 Gb) and *Ambystoma mexicanum* (32 Gb). Based on simulations and backed by analytical derivations, we identify parameter changes originating from increased genome and nuclear size that slow gene expression kinetics. We simulate biological scenarios for which these parameter changes mathematically recapitulate slowed gene expression in *A. mexicanum* relative to *X. laevis*, and we consider scenarios for which additional alterations in gene product stability and chromatin packing are necessary. Results suggest that slowed degradation rates as well as changes induced by increasing nuclear volume and intron length, which remain relatively unexplored, are significant drivers of slowed developmental tempo.

## Introduction

Across the tree of life, genome size and cell volume span a remarkable range, and a positive correlation has been observed between increases in genome size, cell volume, and nuclear volume (Gregory 2001; Sessions 2008; Malerba and Marshall 2021). However, the mechanisms underlying these relationships and the implications associated with such increases remain areas of ongoing research. For example, evolutionary increases in genome, cell, and nuclear size have been found to slow developmental processes (Sessions and Larson 1987; Jockusch 1997; Wyngaard et al. 2005), but the driving mechanisms are poorly understood. Development emerges from the progression and interaction of a wide range of processes taking place at the single-cell level, where increasing genome, cell, and nuclear size impact transcription dynamics, intra-cellular distances, surface area to volume ratios, and other fundamental characteristics (Sessions and Wake 2021; Cadart et al. 2023). We therefore consider how alterations in single-cell processes might translate to slowed developmental tempo as increasing genome, cell, and nuclear sizes change cell structure and functionality.

Vertebrates comprise a large portion of the overall range in genome and cell size across the tree of life, and despite variation in genome size, cell volume, and developmental tempo, many developmental processes remain conserved. Somitogenesis is one such process that is relatively well understood; it is the process through which bilateral pairs of somites, blocks of presomitic mesoderm (PSM) tissue, are patterned along the head-to-tail axis in vertebrate embryos. Somites give rise to the vertebrae, ribs, and musculature of the trunk, among other things. Somitogenesis is typically described using a simplified clock and wavefront model in which cell-autonomous oscillations of a somitogenesis gene (i.e., the “segmentation clock”) interact with Notch, Wnt, FGF, and retinoic acid pathways across the PSM tissue to coordinate proper timing of segmentation of groups of neighboring cells into bilateral pairs of somites (Cooke and Zeeman 1976; Aulehla and Pourquié 2010; Gibb et al. 2010; Klepstad and Marcon 2023). The segmentation clock operates via oscillatory gene expression at the single-cell level, while the wavefront takes place at the intercellular level across the PSM. Vertebrates exhibit species-specific segmentation clocks directly related to oscillatory expression of an autoregulated somitogenesis gene or genes at the single cell level (Matsuda et al. 2020; Diaz-Cuadros et al. 2023; Lázaro et al. 2023). One period of oscillation, i.e., the time required for one cycle of expression, at the single-cell level determines the time needed for a group of neighboring cells to segment off from the larger block of unsegmented PSM tissue, creating a bilateral pair of somites. As a conserved phenomenon that drives developmental tempo while operating at the single-cell level, the segmentation clock is an appropriate lens through which to examine single-cell processes as an underlying link between increasing genome and cell size and slowed development. While species-specific segmentation rates have been linked to biochemical differences at the intracellular level (Matsuda et al. 2020; Rayon et al. 2020; Diaz-Cuadros et al. 2023; Lázaro et al. 2023), the roles of genome and cell size as potential mediators of species-specific gene expression kinetics and therefore developmental tempo have not been explicitly examined.

The oscillation of somitogenesis gene expression at the single-cell level exhibits noise that is damped by cell-to-cell signaling pathways (Keskin et al. 2018), and the segmentation clock tends to slow in the posterior-most somites (Giudicelli et al. 2007; Schröter et al. 2008). General species-specific clock dynamics, however, can still be well-described by the following system of delayed differential equations (DDEs), first proposed by Lewis (2003), which models the segmentation clock by describing coupled oscillatory expression of mRNA and protein


(1) \begin{eqnarray*}
\frac{{dp}}{{dt}} = am\left( {t - {{T}_p}} \right) - bp\left( t \right),\end{eqnarray*}

(2) \begin{eqnarray*}
\frac{{dm}}{{dt}} = f\left( {p\left( {t - {{T}_m}} \right)} \right) - cm\left( t \right),\end{eqnarray*}

where: *p* is protein expression (i.e., the number of protein molecules in a cell); *m* is mRNA expression (i.e., the number of mRNA molecules in a cell); *a* is a rate constant for protein synthesis (protein/mRNA/min); and *b* is a rate constant for protein degradation (minute^−1^) such that *b* ∗ *p*(*t*) is the rate of protein degradation (protein/minute) at time *t*. The rate constant *b* is given by the following expression: *b* = *ln*(2)/*h_p_*, where *h_p_*is the half-life (minutes) of the protein molecule. Similarly, *c* is a rate constant for mRNA degradation given by the following expression: *c* = *ln*(2)/*h_m_*, where *h_m_*is the half-life of the mRNA molecule; and *T_p_*and *T_m_*are the delays associated with protein and mRNA production, respectively. Parameter notation and descriptions are provided again in Table 1.

**Table 1:** Model parameters.

Notation	Description
*p_crit_*	Critical protein threshold, number of protein molecules needed to achieve 10^−9^ M concentration in nucleus
*T_p_*	Delay associated with protein production (min)
*T_m_*	Delay associated with mRNA production (min), sum of:
*T_tx_*	Transcription delay (min)
*T_in_*	Intron splicing delay (min)
*T_exp_*	mRNA export delay (min)
*a*	Rate of protein synthesis (protein/mRNA/min)
*k*	Rate of mRNA synthesis, no repression (mRNA/min/cell)
*b*	Rate constant of protein degradation (min^−1^), equal to *ln*(2)/*h_p_*
*h_p_*	Protein half-life (min)
*c*	Rate constant of mRNA degradation (min^−1^), equal to *ln*(2)/*h_m_*
*h_m_*	mRNA half-life (min)

In Equation (1), describing the rate of change of protein expression, protein synthesis is taken to be linearly dependent on mRNA expression at a time *T_p_*before the current time, *am*(*t* − *T_p_*), and proteins are assumed to degrade by a first-order process, *bp*(*t*). In Equation (2), describing mRNA dynamics, the transcription of mRNA is taken to be controlled by the protein concentration at a time *T_m_*prior to the current time through the Hill function *f*(*p*), given below


(3) \begin{eqnarray*}
f\left( {p\left( {t - {{T}_m}} \right)} \right) = \frac{k}{{1 + {{{\left( {\frac{{p\left( {t - {{T}_m}} \right)}}{{{{p}_{\\smallriptscriptstyle{crit}}}}}} \right)}}^n}}}.\end{eqnarray*}

Here, *k* is the rate constant for mRNA synthesis (mRNA/min/cell) in the absence of repression; *p_crit_*is the number of protein molecules in the nucleus needed to yield an assumed critical concentration of 10^−9^ M within the nucleus associated with transcriptional repression (from Lewis 2003); and *n* is the so-called “Hill coefficient.” The rate of mRNA transcription is dependent upon and inversely related to protein quantity, indicating delayed autorepression. Like Lewis (2003), we assume that these repressive proteins act as dimers and let *n* = 2.

As touched on above, the parameters *T_p_*and *T_m_*account for delays associated with protein and mRNA production, respectively. Delay parameters reflect the reality that biological processes are often a non-instantaneous affair. Mathematically, they distinguish the DDE system above from an ordinary differential equation system and are necessary to generate the sustained oscillations that yield species-specific periods of gene expression (Lewis 2003), corresponding to species-specific rates of somite segmentation.

Here, we adapt Lewis’ model to assess the impact of increasing genome and cell size on segmentation clock period. We use two model amphibian species for which segmentation clock periods and genome sizes are known: the African clawed frog *Xenopus laevis* and the axolotl salamander *Ambystoma mexicanum*, which exhibit a 10-fold difference in genome size. Because both are amphibians, their development proceeds at rates dictated by ambient temperature. Thus, we base our analyses on developmental tempos measured at 20 °C to control for the profound effects of temperature on developmental rates (Pearson and Elsdale 1979; Cossins and Bowler 1987; Bradford 1990). We break delay parameters down into specific transcription, post-transcription, and translation processes, and we incorporate the effects of increasing genome and/or cell size into the appropriate parameters. We also adjust critical protein threshold values to reflect species-specific nuclear volume estimates. Finally, we consider additional potential roles for mRNA and protein stability, which are not directly related to genome or cell size, in the mediation of developmental tempo. We simulate the Lewis model under all of these different scenarios and find that we can reproduce the observed periodicities of the somitogenesis clocks in the two species, but not for all model scenarios. We buttress our simulations with an analytical derivation of the minimal conditions for oscillations in the Lewis model. Our approach suggests how increases in genome size and nuclear volume impact gene expression kinetics to slow developmental tempo. We also reveal potential indirect roles for gene product stability in the mediation of developmental tempo.

## Methods

### Taxon selection

We chose to adapt the Lewis model to *X. laevis* and *A. mexicanum*. Their recorded genome sizes are ∼3.1 Gb (*X. laevis*) and ∼32 Gb (*A. mexicanum*) (Hellsten et al. 2010; Smith et al. 2019), and, although volumes vary across cell types, *A. mexicanum* cell volumes are typically larger than their *X. laevis* counterparts. *Ambystoma mexicanum* nerve cells, for example, are ∼2-times larger than *X. laevis* nerve cells (Roth and Walkowiak 2015), and their red blood cells (RBCs) are ∼10-times larger than in *X. laevis* (Gregory 2024, using cell volume measurements for *A. tigrinum* whose average reported genome size is also ∼32 Gb. Note also that most amphibian RBCs are nucleated). Meanwhile, there is about a three-fold difference in the rate of somite segmentation. In *X. laevis*, bilateral pairs of somites are segmented off from the PSM every 56 min (extrapolated from Pearson and Elsdale (1979)); in *A. mexicanum*, somite segmentation occurs every ∼154 min (Armstrong & Graveson 1988).

### Generating species-specific parameter values

Our first goal was to test if parameter changes directly related to increasing genome, cell, and nucleus size are sufficient to recapitulate the slowed rate of somite segmentation in *A. mexicanum* relative to *X. laevis*. To this end, we started by generating species-specific delay time and critical threshold parameters that capture genome size and nuclear volume, while holding all other parameters (rates of synthesis and degradation) at constant values or ranges between species.

We derived protein and mRNA-production delays, *T_p_*and *T_m_*, by applying estimation methods from Lewis to somitogenesis gene candidates for *X. laevis* and *A. mexicanum*. Vertebrate clock genes are members of the Hairy and enhancer of Split (*Hes/Her*) family of basic helix-loop-helix genes (Kageyama et al. 2007). *Hes/Her* gene family size varies across vertebrates, and the individual cycling members of the clock network also vary (Krol et al. 2011; Eckalbar et al. 2012). In zebrafish and mice, *hes7* has been shown to be the central component of the oscillator (Bessho et al. 2001; Oates and Ho 2002; Holley et al. 2002; Hirata et al. 2004), and its oscillatory pattern has also been shown in the PSM in the lizard *Anolis carolinensis* (Eckalbar et al. 2012). We therefore inferred that *hes7* is the ancestral clock for vertebrates, and that it retains clock function in *A. mexicanum*. In *X. laevis*, in contrast, the *Hes/Her* gene family is greatly expanded to 37 copies, following both ancient allotetraploidization and tandem duplication (Watanabe et al. 2017; Kuretani et al. 2021). The *hes7* orthologs in *X. laevis* are not cyclically expressed in the PSM and, therefore, cannot act as the clock (Jen et al. 1999; Davis et al. 2001; Shinga et al. 2001). In contrast, in *X. laevis*, three *Hes/Her* family genes are known to oscillate in the PSM: *hes5.3, hes5.5*, and *hes5.7* (Li et al. 2003; Blewitt 2009). Of these, the strongest candidate is *hes5.7L* based on experimental data showing that *de novo* protein synthesis is required to repress *hes5.7L* transcription during somite formation, and that *hes5.7L* RNA instability is part of the mechanism underlying its cyclic expression (Davis et al. 2001; Li et al. 2003). *Hes5.7* is absent from the closely related *X. tropicalis* and is thus inferred to be specific to *X. laevis* (Watanabe et al. 2017; Kuretani et al. 2021), suggesting a case of developmental system drift (Haag and True 2021). In *X. laevis, hes5.7L* has a primary sequence length of 1,604 nt; it is made up of 3 exons and 2 introns (lengths: 166 and 113 nt), and its coding sequence is 465 nt (Sayers et al. 2022). In *A. mexicanum, hes7* has a primary sequence length of 8272 nt; it is made up of 4 exons and 3 introns (lengths 3017, 1260, and 2030 nt), and its coding sequence is 783 nt ((Smith et al. 2019) visualized on http://genome.ucsc.edu/).

This information was used to derive species-specific parameters, described below.

*Critical protein threshold, p_crit_*. We calculated the number of protein molecules needed to achieve a critical concentration of 10^−9^ M in the nucleus (as assumed by Lewis (2003)), based on species-specific nuclear volumes. To generate species-specific nuclear volumes, we assumed a spherical nucleus ($V = \frac{4}{3}\pi {{r}^3}$) and estimated species-specific radii of 4 and 5.5 µm using the image processing software Fiji (Schindelin et al. 2012) of stained PSM nuclei in *X. laevis* (Hidalgo et al. 2009) and *A. mexicanum* (Banfi et al. 2012), respectively.

*Protein production delay, T_p_*. We assumed that the delay associated with protein production is equal to translation delay. We estimated species-specific translation delays by applying a translation rate of 6 nucleotides per second (Lewis 2003) to the reported coding sequence lengths.

*mRNA production delay, T_m_*. We considered mRNA-production delay to be a cumulative sum of transcription *T_tx_*, intron-splicing *T_in_*, and mRNA export *T_exp_*delays. We estimated species-specific *T_tx_*values by applying a transcription rate of 20 nucleotides per second (Lewis 2003) to the reported primary sequence lengths. Hoyle and Ish-Horowicz (2013) find that in vivo intron-splicing delay constitutes a relatively constant proportion of ∼8.3% of the overall segmentation clock period in mice, chick, and zebrafish embryos. We applied this proportion to the reported clock periods in *X. laevis* (∼56 min) and *A. mexicanum* (∼154 min) to get species-specific *T_in_*values. We estimated species-specific mRNA export delays, *T_exp_*, using simulations of particle diffusion within a sphere, described in detail below.

*Simulation of mRNA export time parameter, T_exp_*. Before they are released into the cytoplasm, newly transcribed mRNA molecules must journey from their chromatin address to the nuclear periphery where they locate an exit pore. Journeying to the periphery constitutes a relatively large part of this process, on the order of minutes, whereas locating and exporting through a pore once there is relatively rapid, typically on the order of fractions of seconds (Mor et al. 2010; Mor and Shav-Tal 2010; Ben-Yishay and Shav-Tal 2019). mRNA movement through the nucleoplasm takes place via passive diffusion. Both normal and obstructed (sub-) diffusion have been observed (Shav-Tal et al. 2004; Ishihama and Funatsu 2009; Ben-Ari et al. 2010; Mor et al. 2010; Mor and Shav-Tal 2010; Oeffinger and Zenklusen 2012). Normal and obstructed diffusion of RNA can be described by simple and fractional Brownian Motion (fBM), respectively (Mor and Shav-Tal 2010; Oeffinger and Zenklusen 2012; Jeon et al. 2014; Lampo et al. 2017), with obstructed diffusion in the nucleus typically attributed to constrained pathways arising from chromatin organization (Ishihama and Funatsu 2009; Ben-Ari et al. 2010; Mor and Shav-Tal 2010; Oeffinger and Zenklusen 2012; Sheinberger and Shav-Tal 2013). A key difference between fBM and normal Brownian Motion (BM) is the non-independence of increments, or steps, during a fBM random walk (Mandelbrot & Van Ness 1968). In a BM model, step increments are independent, meaning the future of a particle's path is independent of the steps it has already taken on that path.

On the other hand, in an fBM model, step increments are non-independent and negatively correlated (Mandelbrot and Van Ness 1968), causing random walkers or particles to exhibit increased site re-visitation, or backtracking, relative to normal BM. Mathematically, this behavior contributes to the sub-linear relationship between mean squared displacement (MSD) and time associated with sub- or obstructed diffusion. This is in contrast with the linear relationship between MSD and time associated with normal diffusion and a normal BM model (i.e., a particle covers less distance over the same amount of time under an fBM relative to a BM model). Physically, we can imagine molecules backtracking when their trajectories become blocked by a physical obstacle and they must retrace their steps until they find an alternative pathway. We might expect such behavior from mRNA molecules in a chromatin-dense nucleus, for example. Given that both normal and obstructed diffusion of transcripts in the nucleus have been observed, we ran normal and fBM simulations to generate estimates of nuclear export delay, and we compared simulation results.

To generate species-specific estimates for nuclear export, we simulated the (3D) random walk of a diffusing particle within spheres of radii between 3 and 6 µm. This range of radii captures measurements for *X. laevis* and *A. mexicanum* PSM cell nuclei, 4 and 5.5 µm, respectively. We simulated mRNA transcript trajectories and recorded the number of steps needed for our simulated mRNA molecule to first cross the nuclear periphery from the nuclear center. 10,000 trajectories were simulated for each sphere in the specified range (radius of 3–6 with intervals of 0.5).

The transcript trajectory for normal diffusion was simulated by generating *x, y*, and *z* position vectors as cumulative sums of increments (i.e., step sizes) chosen from a normal random distribution. The transcript trajectory for obstructed diffusion was similarly simulated, with the *x, y*, and *z* position vectors generated directly by a fBM function in MATLAB, wfbm, with a Hurst parameter of 0.25. We selected 0.25 as our Hurst parameter based on a basic mathematical definition of obstructed diffusion in 3 dimensions for which MSD is sub-linearly related to time elapsed (*t*)


(4) \begin{eqnarray*}
{{t}^\alpha } = \frac{{MSD}}{{6{{D}_\alpha }}},\end{eqnarray*}

where *α* is the anomalous diffusion exponent, MSD is mean squared distance and *D_α_* is a generalized diffusion coefficient. The exponent alpha is used to describe cases of anomalous and normal diffusion with *α* = 1 corresponding to normal diffusion, 0 *< α <* 1 corresponding to sub-diffusion, and 1 *< α <* 2 corresponding to super-diffusion. The Hurst parameter is typically defined as *H* = *α*/2 (Burov et al. 2011; Sposini et al. 2022). In cells and nuclei where obstructed (i.e., sub-diffusion) diffusion has been observed, alpha values (*α*) have ranged anywhere between 0.3 and 0.8 depending on the cell, molecule type, and observation time (Golding and Cox 2006; Bronstein et al. 2009; Weber et al. 2010 as cited in Burov et al. 2011). Lacking observations for mRNA in amphibian developmental systems, we selected *α* = 0.5 as a plausible value that reasonably lies within the range of values observed in other systems, leaving us with the Hurst parameter *H* = 0.25.

For both simulations, the number of steps required by a particle trajectory to first exit the domain was retrieved and averaged. We assumed that each step takes one second, and we scaled the particle trajectory such that the estimated nuclear export time for a radius of 3 µm (our chosen lower limit) would agree with previous empirical results. The lower limit of our chosen range of radii corresponds to an estimate of nuclear radii in zebrafish PSM cells made using the image processing software Fiji (Schindelin et al. 2012) and images of stained nuclei across the PSM in zebrafish from Keskin et al. (2018). Empirical observations in Hoyle and Ish-Horowicz (2013) suggest a 3.36 min mRNA exit time associated with *her1*(*hes7*), a somitogenesis gene in zebrafish. We therefore scaled our trajectory time scales in both simulations such that it takes, on average, about 202 seconds, that is, 3.36 min (corresponding to 202 steps of the simulation) to first exit a sphere of radius 3 µm.

We assumed the nuclear center to be the initial mRNA position based on known chromosomal territories associated with the somitogenesis gene in humans and mice coupled with patterns of synteny observed across vertebrates. That is, in both mice and humans, the somitogenesis gene, *hes7*, is found on gene-rich chromosomes 11 and 17 (which are homologs), respectively; both chromosomes localize in their respective nuclear centers (Boyle et al. 2001; Kile et al. 2003; Mayer et al. 2005; Zody et al. 2006). Broad patterns of synteny conservation and topologically associated domain conservation have been observed across vertebrates (Smith et al. 2019; Schloissnig et al. 2021), suggesting that this pattern extends beyond humans and mice.

*Analytical results for the mean first exit time*. The time needed, on average, for an mRNA molecule to exit the nucleus from its center can be thought of as a mean first exit time (MFET) problem. Analytical results exist for the MFET from the center of a sphere under normal diffusion (Gitterman 2004; Redner 2022) and are given by


(5) \begin{eqnarray*}
\mathit{MFET} = \frac{{{{r}^2}}}{{6D}}.\end{eqnarray*}

Given the measured nuclear export time of 3.36 minutes for *her1* in zebrafish and an estimated nuclear radius of 3 µm, we assumed a diffusion coefficient *D* of 0.45 µm^2^/min to generate analytical nuclear export time estimates across increasing radii under normal diffusion. We show this along with simulation results in Fig. 3. An analogous analytical result for the sub-diffusive case does not exist (Gitterman 2004; Yuste and Lindenberg 2004). This is why stochastic simulations, which are also intuitively more appealing, were used to estimate nuclear export times.

*Parameters held constant*. For the first set of analyses, we held the rates of mRNA production (in the absence of inhibition), protein production, and mRNA degradation constant, at *k* = 33 mRNA/min, *a* = 4.5 protein/mRNA/min, and *c* = *ln*(2)/3 min^−1^ corresponding to a half-life *h_m_* = 3 min (Lewis 2003). We considered a range of protein stabilities or degradation rates *ln*(2)/22 ≤ *b* ≤ *ln*(2)/3 min^−1^, corresponding to half-lives of 3 ≤ *h_p_* ≤ 22 min. This range was chosen based on typical reported and estimated protein stability of the somitogenesis gene across model vertebrates, namely zebrafish and mice. We tested the model across a range of protein half-lives due to its important role in mediating the period of gene expression (Lewis 2003; Hirata et al. 2004; Takashima et al. 2011; Matsuda et al. 2020; Lázaro et al. 2023) (see also Table 3: Sensitivity Analysis). We emphasize that, although experimental perturbations of cell size as well as analyses of cell volume changes throughout the cell cycle reveal that synthesis and degradation rates scale with cell volume (Sun et al. 2020; Berry et al. 2022; Basier and Nurse 2023; Swaffer et al. 2023), our comparison is between two taxa with evolved differences in target cell size, and the effects of such evolved differences on biosynthesis remain unknown. Thus, holding these parameter values constant is a logical starting assumption.

### Extrapolation of periodicity to compare with known somite segmentation rates

For our first set of analyses, we plugged the parameter values described above back into the Lewis model, and we used the DDE solver ddesd in MATLAB to generate solutions across identical ranges of protein stability *h_p_*and species- and diffusion-specific ranges of total delay time, *T_m_* + *T_p_*. We assessed the period of gene expression emerging for each set of solutions, and we compared it to the known somite segmentation rate of the corresponding species. In doing so, we wanted to first verify that our parameter selection does in fact yield the correct period of oscillation for *X. laevis*, after which we could determine if parameter changes directly driven by genome and cell size differences are sufficient to capture slowed developmental rate in *A. mexicanum* relative to *X. laevis*.

To assess periodicity, we created vectors to store the local extrema (i.e., local minimum and maximum values) and corresponding time stamps for each solution. Gene expression tends to spike in the first 4–5 cycles of oscillation before settling into a long-term pattern, so we removed the first 5 cycles of oscillation from our data to avoid skewing. The period of oscillatory gene expression was calculated by taking the average difference between successive time stamps associated with local minima (using local maxima would yield the same results). We did this across a time span of 0–3100 min. The time span, 3100 min, was chosen based on the fact that (at least) 20 somites are observed in *A. mexicanum* embryos, each requiring ∼154 min to form (Armstrong and Graveson 1988). This time span also works well for *X. laevis*, with approximately 50 somites (Dali et al. 2002), each taking ∼56 min to form (Pearson and Elsdale 1979). For both species, oscillations must remain robust throughout the set time span. We defined robust oscillations as having an amplitude (the height of an oscillation, or difference between local minimum and maximum) of no fewer than 10 molecules throughout the time span. If this requirement was not met, the oscillations were considered damped and the periodicity was defined as Inf (infinite). We chose 10 molecules as a conservative finite cut-off based on observed average RNA transcript amplitudes of zebrafish segmentation clock genes *her1* and *her7* which are ∼41 and ∼49 molecules, respectively (Keskin et al. 2018). However, when we scale the cut-off amplitude for the *A. mexicanum* models with the increase in estimated nuclear volume relative to *X. laevis* (i.e., scaling to 26 mRNA molecules to reflect a ∼2.6-fold increase in volume), results do not appreciably change (results not shown).

The assessment of periodicity described above was done for both mRNA and protein counts, and the difference in periodicity was always within 0.2 min (see Supplemental Material 1). In other words, results are relatively similar for both sets of oscillations, so we chose mRNA periodicity to represent overall system behavior.

### Calculating average amplitude of expression

We also calculated the average amplitude of mRNA expression across the parameter combinations in each model. To do this, we used the extrema vectors described above to create an additional vector that stores the difference between local minima and maxima corresponding to every complete cycle of oscillation, excluding data from the first 5 oscillation cycles that were cut out. The resulting vector gives the amplitude associated with each complete oscillation cycle. We averaged over all vector entries to get an average amplitude associated with a particular parameter combination. For all combinations with periodicity defined as Inf (infinite), we set amplitude equal to 0, to keep results consistent with each other.

### Sensitivity analysis

In Supplemental Material 2, we derive general conditions for the emergence of oscillations for the Lewis Model. However, analytical solutions of the Lewis model equations for arbitrary parameters are not known. Therefore, we also assessed the impact of individual parameter changes on the period of gene expression using a sensitivity analysis.

To assess the impact of individual parameter changes on the period of gene expression, we increased and decreased each individual parameter (using total delay as opposed to taking *T_p_*and *T_m_*individually) by 50% while holding all other parameters constant. For each resulting parameter combination, we calculated the resulting period of oscillation. We chose to vary parameter values by 50% to account for a relatively high level of uncertainty in our parameter selections. Our starting set of parameters corresponds to an *A. mexicanum* BM model with protein half-life arbitrarily set at *h_p_* = 15 min. Each individual parameter was increased and decreased by 50% from its starting value (*a* = 4.5, *k* = 33, *p_crit_* = 420, *h_m_* = 3, *h_p_* = 15, Total delay *T_m_* + *T_p_* = 33.82), while all others were held at their respective original values. The resulting changes in period of gene expression were tracked.

### Testing whether scaling mRNA stability with export time yields the rate of somite segmentation in *A. mexicanum*

Our mRNA export simulations suggest that transcripts take much longer to leave larger nuclei. We therefore assessed the impact of scaling mRNA stability with estimated nuclear export on the *A. mexicanum* segmentation clock period. To this end, we re-considered both *A. mexicanum* models, normal and fBM corresponding to normal and obstructed diffusion, under 3 different levels of mRNA stability: half-life equal to mRNA export time, half-life equal to 50% of mRNA export time, and half-life equal to 25% of mRNA export time. While holding all other *A. mexicanum* species- and diffusion-specific parameters at their initial values (Table 2), we re-generated solutions and assessed periodicity for the Lewis model given *h_m_* = *T_exp_, h_m_*= ½ *T_exp_*, and *h_m_* = ¼ *T_exp_*.

**Table 2:** Initial parameter values.

		Species-specific values		
Parameter		*X. laevis*	*A. mexicanum*	Genome size or nuclear volume captured?
*p_crit_*		161	420	Increasing nuclear volume
*T_p_*		1.29	2.18	Neither
*T_tx_*		1.34	6.89	Increasing genome size
*T_in_*		4.65	12.78	Neither
*T_exp_*	*BM model*	6.39	11.97	Increasing nuclear volume
	*fBM model*	8.36	26.27	Increasing nuclear volume
*T_m_*	*BM model*	12.38	31.64	Increasing genome size and nuclear volume
	*fBM model*	14.35	45.94	Increasing genome size and nuclear volume
**Parameter**	**Values and ranges held constant across species and diffusion-type**			
*a*	4.5			
*k*	33			
*c*	*ln*(2)/*h_m_*, where *h_m_* = 3			
*b*	*ln*(2)/*h_p_*, where 3 ≤ *h_p_* ≤ 22			

### Generalizing mRNA export simulations

We generalized our mRNA export simulations (described above) across a range that encompasses observed values across the tree of life: nuclei with radii ranging from ∼0.5 to 13 µm (see Supplemental Material 3), based on the minimum and maximum nuclear volumes reported in the dataset used by Malerba and Marshall (2021) (https://doi.org/10.5061/dryad.vq83bk3ss) while assuming a spherical volume $V = \frac{4}{3}\pi {{r}^3}$. We also ran simulations for which the initial position is selected from a uniform distribution as opposed to being set at the origin (representing the nuclear center). The uniform distribution we drew initial *x, y*, and *z* positions from encompass a domain that is $\frac{3}{4}$ of each radius. Thus, initial positions could occur throughout the theoretical nucleus, excluding the periphery. We chose to exclude the nuclear periphery because this is where heterochromatin is spatially concentrated and reduced transcriptional activity has been observed (Bizhanova and Kaufman 2021).

## Results

### Period of gene expression is most sensitive to changes in delay and stability parameters

Conditions for the emergence of oscillations for simplified versions of the Lewis model, assuming a single degradation rate and a single delay, have been obtained analytically (Verdugo and Rand 2008), but it was not clear whether the results would generalize. We therefore derived conditions for the emergence of oscillations for the full Lewis Model (see Supplemental Material 2). We found that oscillating solutions require (i) the geometric mean of the degradation rates $\sqrt K $ to be less than an upper bound, and (ii) total delay *T_m_* + *T_p_*to be equal to or greater than a critical value, *T_crit_*. Both $\sqrt K $ and *T_crit_*depend upon the kinetic constants of the model. In particular, *T_crit_*is positively correlated with *h_p_*, the protein half-life (Supplemental Material 2). Thus, sustained oscillations with longer-lived proteins require longer delay times.

We use the sensitivity analysis to track the specific impact of individual parameter changes on the period of gene expression, the results of which are shown in Table 3. In agreement with results in Lewis (2003), we find that the period of gene (mRNA) expression is most sensitive to changes in total delay, *T_m_* + *T_p_*, and stability, *h_m_*and *h_p_*, parameters and is relatively insensitive to changes in *a, k*, and *p_crit_*parameters. This result suggests that uncertainty in parameter values for *a, k*, and *p_crit_*are unlikely to significantly impact model results and supports our choice to hold *a* and *k* constant across species. Although *p_crit_*also has no significant impact on periodicity of gene expression, we introduce species-specific values for *p_crit_*in order to complement the nuclear radii estimates used in nuclear export simulations.

**Table 3:** Sensitivity analysis.

Parameter changed	Change in period resulting from 50% increase	Change in period resulting from 50% decrease
*a*	<1% change	<2% change
*k*	<1% change	<2% change
*p_crit_*	<1% change	<2% change
*h_m_*	∼5% change	Damped/no oscillations
*h_p_*	Damped/no oscillations	∼10% change
Total delay (*T_m_* + *T_p_*)	∼40% change	Damped/no oscillations

Results from our sensitivity analysis also reveal that when protein is too stable and total delay time is too low, robust oscillations do not emerge. In other words, total delay must be relatively large compared with protein half-life for oscillations to emerge, in agreement with our analytical results and previous mathematical and empirical results (Lewis 2003; Hirata et al. 2004; Takashima et al. 2011).

### Parameter changes directly linked to increasing genome and cell size can mathematically recapitulate slowed developmental tempo

Using simulated export times, we generate species- and diffusion-specific general delay times associated with mRNA production, a sum of transcription, intron splicing, and nuclear export delays. All other parameters are held constant either across species (*a, k, b, c*) or at species-specific values across diffusion models (*p_crit_, T_p_*). All of these values are shown in Table 2.

**Fig. 1:**
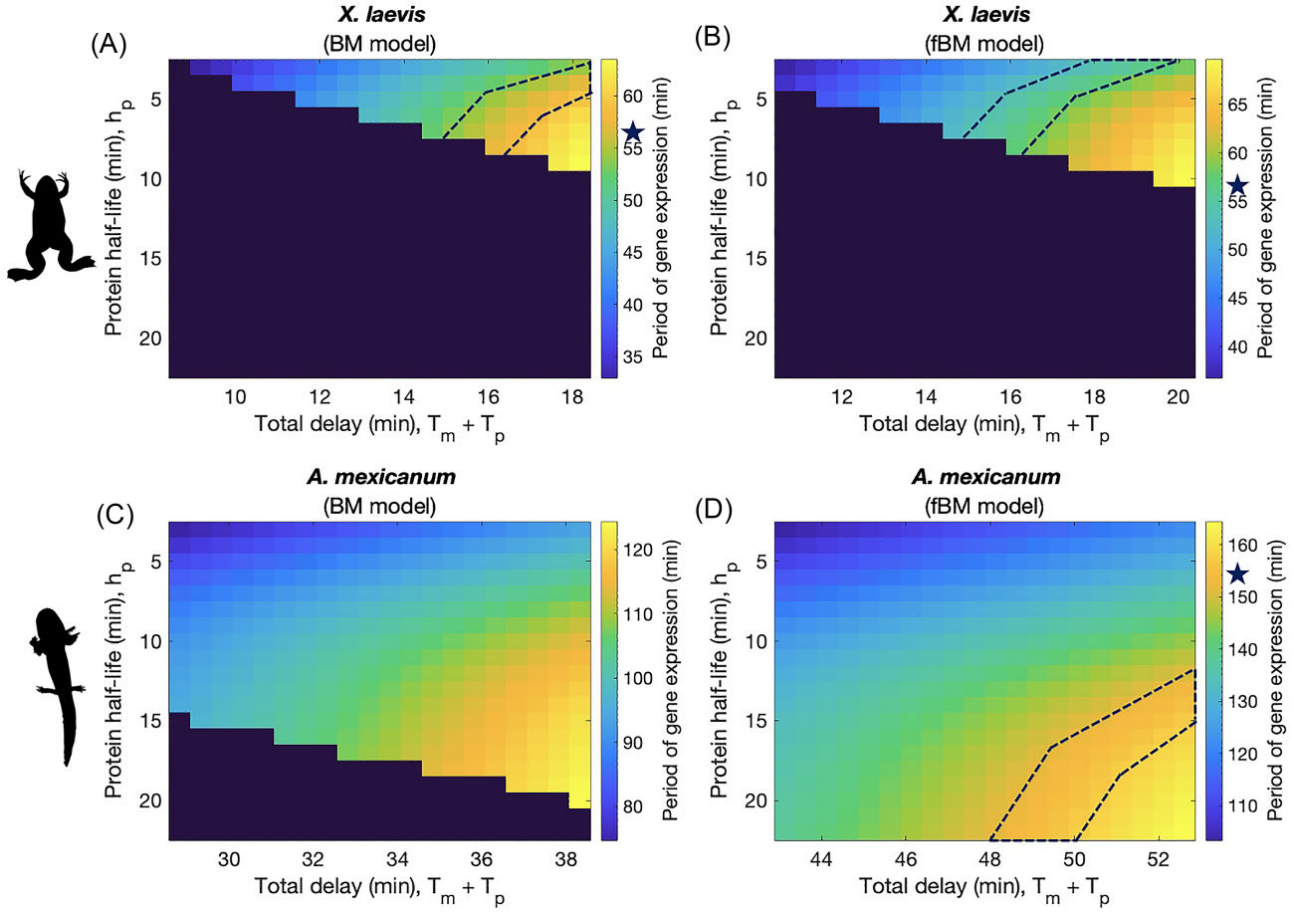
Resulting periods of gene expression given for each species- and diffusion-specific model. Each combination of protein half-life and total delay time corresponds to a period shown in the colorbar to the right of each model plot. Dark purple areas are non-oscillatory; stars show where the known species-specific rates of somite segmentation are found on the colorbar, and the corresponding regions are outlined in dashed lines. Results for: (A) *X. laevis* model when normal diffusion is assumed/Brownian Motion is modeled; (B) *X. laevis* model when obstructed diffusion is assumed/fractional Brownian Motion is modeled; (C) *A. mexicanum* model when normal diffusion is assumed/Brownian Motion is modeled; and (D) *A. mexicanum* model when obstructed diffusion is assumed/fractional Brownian Motion is modeled.

The resulting periods of oscillation for each model are shown in Fig. 1 (with non-oscillatory combinations/regions, assigned period Inf, shown in dark purple).

We test across a range of parameter combinations: on the x-axis, we have values of total delay, *T_m_* + *T_p_*, that fall within ± 5 minutes of the species- and diffusion-specific values given in Table 2; on the *y*-axis, we have a range of protein stability corresponding to 3 ≤ *h_p_* ≤ 22 min. The observed rate of somite segmentation in *X. laevis* (∼56 min) is captured by a subset of total delay and protein stability combinations under both normal and sub-diffusive (obstructed diffusion) conditions; this subset is outlined in the plot by a dashed-line in Fig. 1A and B. In confirming that we can achieve the correct period of oscillation for *X. laevis*, the results in Fig. 1A and B provide support for our methods of parameter estimation; these results also act as a plausible baseline against which the *A. mexicanum* models can be compared. Meanwhile, the observed rate of somite segmentation in *A. mexicanum* (∼154 min) is only captured under sub-diffusive conditions. Genome and nucleus size-driven increases in delay time and concentration threshold are sufficient to fully recapitulate slowed development in *A. mexicanum* when nucleoplasmic movement of transcripts is assumed to be sub-diffusive (Fig. 1D), but are insufficient when normal diffusion is assumed (Fig. 1C). When normal diffusion is assumed, genome and cell size-driven parameter changes can slow the period of oscillatory gene expression down to ∼125 min, 29 min faster than the known rate of somite segmentation. The additional delay introduced by assuming sub-diffusion, about 14 min longer, is needed to produce a set of total delay and protein stability combinations that yield a ∼154 min period of oscillation.

### mRNA export is two- to three-fold slower in *A. mexicanum* relative to *X. laevis*

mRNA export time roughly doubles in *A. mexicanum* relative to *X. laevis* when normal diffusion is assumed, and roughly triples when obstructed diffusion is assumed (see *T_exp_*in Table 2). The impact of obstructed diffusion on export time becomes more pronounced (i.e., the gap between export times for the two diffusion types becomes wider) as nuclear radius and therefore volume increases (see Fig. 3). Analytical and simulation results for nuclear export times under normal diffusion agree relatively well.

### Scaling mRNA stability with nuclear export time yields biologically plausible recapitulation of slowed developmental tempo

Although the fBM model shown in Fig. 1D yields the known rate of somite segmentation in *A. mexicanum* based solely on genome and cell size differences in parameter values, an additional increase in mRNA stability seems logically necessary given our simulated nuclear export times. The models shown in Fig. 1 assume an mRNA degradation rate associated with a half-life of 3 min. However, we are working with simulated mean export times of ∼12 and ∼26 min in *A. mexicanum* PSM nuclei under normal and sub-diffusive conditions, respectively. Under these assumptions, a vast majority of mRNA molecules are expected to degrade long before ever leaving the nucleus.

We therefore test if the *A. mexicanum* segmentation clock can also be recovered when mRNA stability is increased relative to export time such that a greater proportion of transcripts are able to exit the nucleus before degrading. Although it is established that some fraction of RNA transcripts will degrade in the nucleus before exiting into the cytoplasm, the extent of degradation seems to differ across transcript types and remains relatively understudied (Smalec et al. 2022). Our parameter estimates for *X. laevis* yield the following patterns: under normal diffusion, mRNA half-life (3 min) is ∼47% of mRNA export delay (6.32 min), and under obstructed diffusion, mRNA half-life (3 min) is ∼36% of mRNA export delay (8.41 min). In terms of degradation, ∼76% of mRNA transcripts would be expected to degrade before leaving the nucleus under normal diffusion. Under obstructed diffusion, ∼82% of mRNA transcripts would be expected to degrade before leaving the nucleus. Meanwhile, when we compare the typical estimate for mRNA half-life in *Danio rerio*, also 3 min, to the reported in vivo export time, 3.36 min (Hoyle and Ish-Horowicz 2013), we see that mRNA half-life is 83% of export time. This corresponds to an expectation that ∼58% of mRNA transcripts would degrade before leaving the nucleus. The three potential scaling scenarios for mRNA stability and nuclear export time in *A. mexicanum* that we explore are within the range of these empirical and theoretical estimates. We first set mRNA half-life equal to diffusion-specific export times, *h_m_* = *T_exp_*, implying that 50% of mRNA transcripts degrade before leaving the nucleus; we then set mRNA half-life equal to 50% of the simulated export times, *h_m_*= ½ *T_exp_*, implying that 75% of mRNA transcripts degrade before leaving the nucleus; finally, we set mRNA half-life equal to 25% of the simulated export times, *h_m_*= ¼ *T_exp_*, implying that 87.5% of mRNA transcripts degrade before leaving the nucleus.

**Fig. 2:**
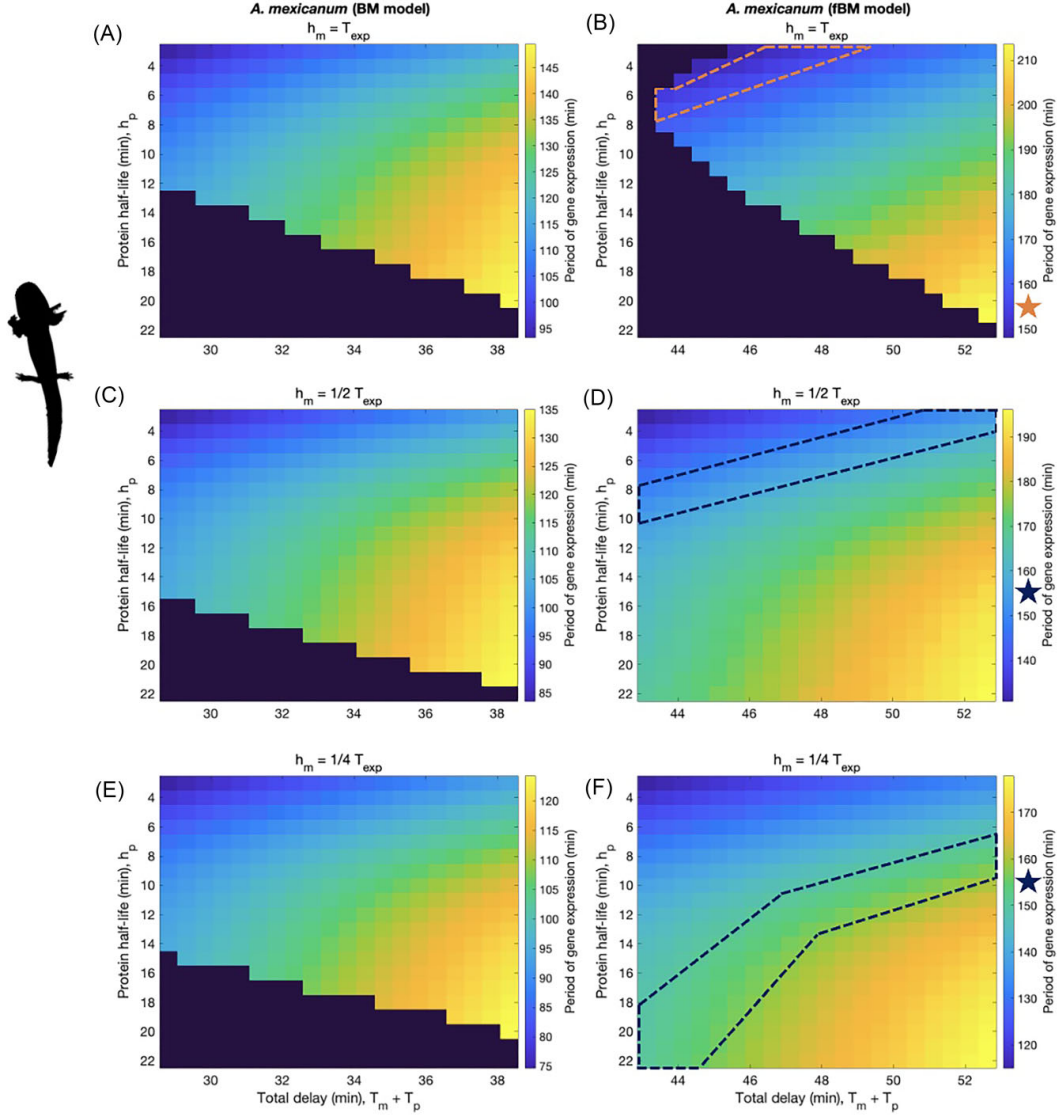
Resulting periods of gene expression for *A. mexicanum* models: (A), (C), and (E) normal diffusion/Brownian Motion; (B), (D), and (F) obstructed diffusion/fractional Brownian Motion. mRNA half-life is held constant at: (A) and (B) diffusion-specific estimates for mRNA export delay; (C) and (D) half of estimated mRNA export delays; (E) and (F) a quarter of estimated mRNA export delays. ^∗^Note: Color of star/outline is chosen for contrast and has no additional meaning.

In Figure 2, we show plots of *A. mexicanum* model results under both normal and fractional BM conditions with these three increased levels of mRNA stability. When normal diffusion of transcripts through the nucleoplasm is assumed, Fig. 2A, C, and E, none of the mRNA stability scenarios produce a subset of protein half-life and total delay time parameter values that yield a period of 154 min. Under obstructed diffusion, Fig. 2B, D, and F, all mRNA stability scenarios produce a subset of protein half-life and total delay combinations that fully yield a period of ∼154 min, capturing the known rate of somite segmentation in *A. mexicanum*.

**Fig. 3:**
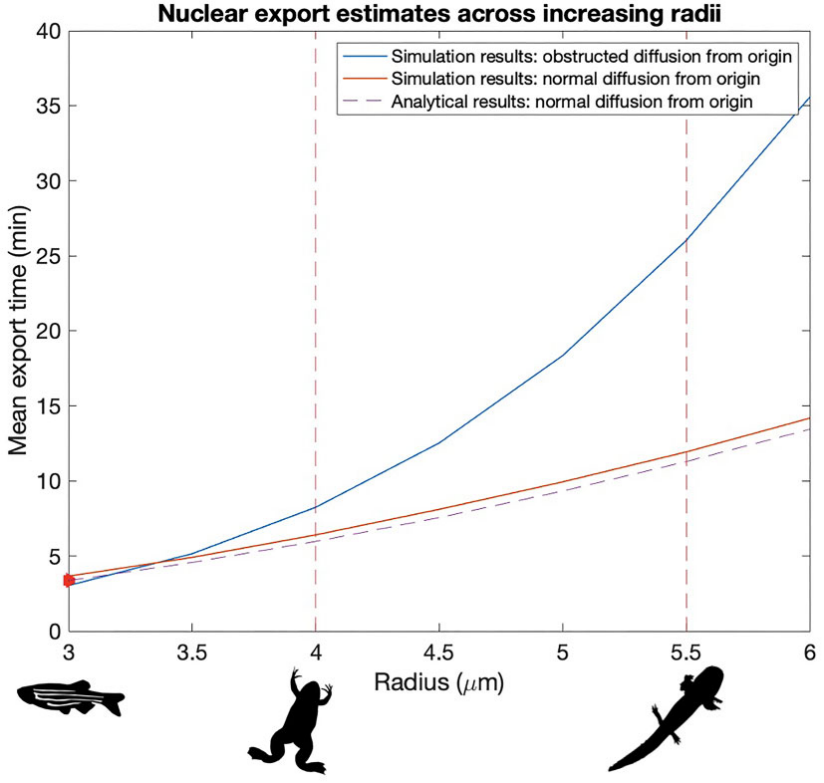
Nuclear export estimates under normal and obstructed diffusion across a range of radii that captures estimates for *X. laevis* and *A. mexicanum* PSM nuclei (shown by the red dashed lines). An initial position at the nuclear center is assumed. Trajectories are scaled such that a radius of 3 µm corresponds to a mean export time of ∼3.36 min (shown by the red arrow) to match the reported export time of *her1 (hes7)* in zebrafish (Hoyle and Ish-Horowicz 2013). Analytical results for the normal diffusion case are also shown. ^∗^Note: There is a limit to how closely 3 µm can be scaled with ∼3.36 min. As a result, obstructed diffusion mean export times start off slightly faster than normal diffusion when radius *r <* 3.5 µm, but this is not necessarily biologically meaningful, and mean export times are quick to converge back to expectations.

## Discussion

Previous research on developmental timing has pointed toward intron length and differences in biochemical characteristics, like degradation rates and network delays, as sources of species-specific tempo (Swinburne et al. 2008; Swinburne and Silver 2008; Matsuda et al. 2020; Rayon et al. 2020; Lázaro et al. 2023). In our study, we not only reconsider these points while contextualizing them within the scope of increasing genome and cell size, but we also make spatially explicit considerations that set our study apart from others.

In Table 2, we outline how species-specific parameters differ between *X. laevis* and *A. mexicanum*, and we note whether each change (based on our methods of estimation) captures increases in genome size or nuclear volume, or neither. Using these species- and diffusion-specific parameters, we first verified that our parameter estimates for *X. laevis* were able to recapitulate the ∼56 min segmentation clock. This indicated that our methods of parameter estimation were reasonable, and we moved forward using the same methods for our *A. mexicanum* models, testing for parameter changes and combinations that recapitulate the ∼154 minute segmentation clock. The results of our models reveal the following potential mechanisms through which developmental tempo slows with increased genome and nuclear size.

### Increasing intron length slows developmental tempo through transcriptional delays

Increasing the sum of delays in the gene regulatory network has the most significant impact on the period of gene expression (see Table 3: Sensitivity Analysis). When we break total delay down into individual components, we see that while transcriptional delay, *T_tx_*, is not the most significant contributor to total delay, *T_m_* + *T_p_*, it confirms an intuitive link between increasing genome size and the alteration of oscillatory gene expression kinetics. This is because transcriptional delay includes the time needed to transcribe the entire primary gene sequence, including intronic regions, which scale positively with genome size. Indeed, increasing intron size (in part due to transposable element insertions) is a major driver of genomic expansion in *A. mexicanum* (Nowoshilow et al. 2018), and while intronic regions only constitute ∼17% of the *hes5.7L* primary sequence in *X. laevis* (279 bp of intronic sequence), they make up ∼76% of the *hes7* primary sequence in *A. mexicanum* (6307 bp of intronic sequence). This is a general pattern observed across orthologous genes in *A. mexicanum* and *X. laevis* (Nowoshilow et al. 2018). Interestingly, this pattern of intronic expansion seems to be constrained in developmental genes, where an ∼11-fold increase in average intron length is observed between *X. laevis* and *A. mexicanum* in contrast with an almost 20-fold increase seen in non-developmental genes (Nowoshilow et al. 2018). In Nowoshilow et al. (2018), the role of transcription in developmental timing is suggested as an explanation for this pattern. The time required to transcribe intronic regions of genes (i.e., “intron delay”) has not only been identified as a potential mediator of developmental timing via its role in gene regulatory networks (Swinburne and Silver 2008), but has also been suggested to link genomic gigantism in particular with slowed development and regenerative abilities through its impact on gene expression kinetics (Sessions and Wake 2021).

However, according to our computational results, increased transcriptional delay alone cannot drive the vast differences in overall delay time between *X. laevis* and *A. mexicanum*. Previous work experimentally increasing intron length in oscillatory genes in mice also showed negligible impact on oscillatory period, although this negative result likely reflected the target gene (*Lnfg*) being outside of the core oscillator (Stauber et al. 2012; Bochter et al. 2022). Given the limited effects of transcriptional delay on overall delay time, our model therefore also implies that intron delay alone cannot drive slowed developmental tempo observed in the *A. mexicanum* segmentation clock relative to that of *X. laevis*. Instead, we see in Table 2 that the delay associated with increasing nuclear volume, *T_exp_*is the most significant contributor to overall delay time across all species- and diffusion-specific models. We note that our inference of candidate clock genes, although justified phylogenetically, has not been confirmed experimentally; these results suggest that our conclusions would be more robust to error in candidate gene length than chromatin address. It is also worth noting that although the delay associated with intron-splicing, *T_in_*is an important contributor to total delay, and the difference between species-specific values for *T_in_*is in fact larger than that between species-specific values for *T_tx_*, inter-species differences in splicing kinetics cannot be clearly linked with differences in intron or, by extension, genome size (Khodor et al. 2012).

### mRNA export time increases with nuclear volume and has a pronounced impact on developmental tempo

Increasing nuclear volume between *X. laevis* and *A. mexicanum* is captured by the critical protein threshold value, *p_crit_*, and mRNA export delay, *T_exp_*, parameters. The increase in critical protein threshold accounts for the fact that in a larger nucleus, more protein molecules are needed to reach the same critical concentration of 10^−9^ M required for transcriptional repression to act “in earnest” (Lewis 2003). However, changes to *p_crit_*have a very limited impact on the period of gene expression relative to other parameter changes. Meanwhile, the increase in mRNA export delay in *A. mexicanum* relative to *X. laevis* has a relatively pronounced impact on the period of gene expression.

Our simulations position mRNA export as the largest contributor to total delay time. This is consistent with observations that find mRNA export delays to be longer than both transcriptional and intron splicing delays in mice, chick, and zebrafish segmentation clocks (Hoyle and IshHorowicz 2013). As the largest contributor to total delay time, mRNA export is also the largest contributor to overall differences in *X. laevis* and *A. mexicanum* segmentation clocks, implying that spatially induced delays in gene regulatory networks play a significant albeit underexplored role in the slowing of cellular and developmental processes across increasing cell and nuclear volumes. Although the increase in the estimated radius of PSM nuclei in *A. mexicanum* relative to *X. laevis* may seem minimal, 5.5 µm compared with 4 µm, simulation results demonstrate that mRNA export delay still nearly doubles in *A. mexicanum* when normal diffusion is assumed (in agreement with analytical MFET results for normal BM) and nearly triples when obstructed diffusion is assumed.

Lázaro et al. (2023) compare segmentation clock periods across six species of mammals *in vitro* using induced PSM cells and reveal a ∼two-fold difference in cell volumes; our results suggest that associated differences in nucleus size and mRNA export times could also be contributing to the observed differences in segmentation clock periods.

The impact of increasing radius on export time, particularly assuming obstructed diffusion, becomes more pronounced when the range of radii is expanded past an upper limit of 6 µm (see Supplemental Material 3), suggesting that the nuclear center in species with the largest known genome sizes may have become “uninhabitable” for genes with dynamic expression patterns. If we introduce transcripts whose positions are drawn from a uniform distribution within the nucleus, as opposed to always starting from the origin/nuclear center, the impacts of increasing radius and obstructed diffusion on mean export time are reduced. The distribution of export times becomes more skewed toward lower values (see Supplemental Material 3) likely because most transcript trajectories start closer to the periphery, relative to the nuclear center, when initial positions are chosen from a uniform distribution. In very large nuclei, we hypothesize that genes—especially those with dynamic expression patterns—may be constrained to occupy these locations away from the nuclear center, suggesting an overall effect of genome expansion on the organization of chromosomal territories.

### Simulations position increasing chromatin density as a driver of slowed development

Across the tree of life, decreasing nucleus to cell volume ratios are observed as cells increase in volume (Malerba and Marshall 2021). In other words, larger cells have relatively smaller nuclei, though absolute nuclear volumes still increase with cell volume. Given the well-established positive correlation between genome size and cell volume, we might also state that nuclei become relatively smaller with increasing genome size. As a result, we would see an increasing amount of genetic material packed within relatively smaller nuclei, implying greater chromatin packing density as genome size and absolute nuclear volumes increase. Readers will recall that chromatin structure density is a commonly cited source of obstructed diffusion observed in the nucleus (Ishihama and Funatsu 2009; Ben-Ari et al. 2010; Mor and Shav-Tal 2010; Oeffinger and Zenklusen 2012; Sheinberger and Shav-Tal 2013), meaning that we would expect to see obstructed diffusion of transcripts in absolutely larger nuclei/organisms with relatively large genomes. In our model, we capture chromatin density in our obstructed diffusion model, but we note that since we choose our Hurst parameter *H* = 0.25 based on observations made in model cell lines, it's also possible that our model underestimates nuclear export times for larger nuclei if they are in fact more crowded.

With these empirical possibilities in mind, we turn toward our theoretical results. In Fig. 1A and B, we see that both diffusion models are able to fully recapitulate the *X. laevis* segmentation clock, while obstructed diffusion (modeled by fBM) must be assumed to fully recapitulate the slowed *A. mexicanum* segmentation clock (shown in Fig. 1D). Similarly, in Fig. 2, we have that obstructed diffusion still must be assumed, regardless of mRNA stability, in order to recapitulate the slowed *A. mexicanum* segmentation clock. Meanwhile, under normal BM, all model scenarios fall short of fully recapitulating the *A. mexicanum* segmentation clock. Further increases in protein and/or mRNA stability do not resolve this issue (see Supplemental Material 4), suggesting that normal diffusion does not introduce sufficient delays into the system. These results indicate that obstructed diffusion is sufficient but not necessary to recapitulate the segmentation clock when genome size is relatively small (*X. laevis*) yet becomes both sufficient and necessary when genome size is relatively large (*A. mexicanum*), consistent with the hypothesis that chromatin packing density increases with genome size. Somitogenesis transcripts must exhibit obstructed diffusion in the nucleoplasm of *A. mexicanum* in order to slow the segmentation clock to an appropriate pace, thus positioning increasing chromatin density as a potential driver of slowed development.

### Gene product stability also acts to mediate developmental tempo

Focusing on Fig. 2B, D, and F (fBM model results), we can see that as mRNA stability changes, so too does the range of protein half-life and total delay time combinations yielding a gene expression period of 154 min. This is in agreement with previous studies that point to gene product stability as a mediator of species-specific segmentation clock periods (Hirata et al. 2004; Matsuda et al. 2020; Rayon et al. 2020; Lázaro et al. 2023). However, some mRNA stability scenarios yield more reasonable results than others. In Fig. 2B and D, with mRNA half-lives of 26.27 and 13.14 min, the 154 min period of oscillation is captured for protein half-lives that range (approximately) from 3 to 8 and 3 to 11 min, respectively. As a result, we would have to assume that protein is about as stable or less stable than mRNA, which is inconsistent with reported patterns for Hes7 in mice and humans (Matsuda et al. 2020). In Fig. 2F, for which mRNA half-life is set to 6.54 min, we get a larger subset of parameter combinations that yield a period of 154 min. Across this subset, protein half-lives range from being only slightly more stable than mRNA at ∼8 min to almost 4-times as stable as mRNA at ∼22 min. The resulting relationship between mRNA and protein stability—that protein molecules exhibit increased stability relative to their transcripts—is more consistent with the patterns reported in Matsuda et al. (2020). However, setting *h_m_* = ¼*T_exp_* implies that a vast majority of mRNA transcripts, 87.5%, will degrade before exiting the nucleus. We might expect mRNA amplitude (i.e., number of transcripts in the cell) to be extremely low under this scenario, but this is not the case. In fact, mRNA amplitudes estimated under the fBM/obstructed diffusion case when *h_m_* = ½*T_exp_* (13.14 min) or ¼*T_exp_* (6.54 min) range from 40 to 350 molecules (see Supplemental Material 5). These parameter estimates are broadly consistent with empirically estimated amplitudes of 41 and 49 molecules for the zebrafish somitogenesis genes *her1* and *her7*; accounting for differences in nuclear (and by proxy, cell) volumes, mRNA amplitudes of 300 molecules in *A. mexicanum* achieve concentrations similar to those empirically measured in zebrafish.

Overall, our results suggest an interesting tradeoff between the possible mRNA stability scenarios described above: in order to mathematically recapitulate the slowed *A. mexicanum* segmentation clock, we must either assume that a vast majority of mRNA transcripts degrade within the nucleus, or that the protein molecules in this system are less stable than their transcripts. The huge nuclear volume, coupled with the assumption of obstructed diffusion, necessitates one of these two possible deviations from conventional gene expression kinetics as genome and nuclear size increase from those seen in *X. laevis* to those seen in *A. mexicanum*. Based on reported patterns for Hes7 in mice and humans (Matsuda et al. 2020), as well as our mRNA amplitude results (for the fBM case, specifically), we propose that widespread nucleoplasmic degradation of the somitogenesis transcript is more plausible than transcripts that are more stable than the corresponding protein molecules.

What remains the same across increasing genome size and nuclear volume is that, as shown in previous implementations of the Lewis model (Hirata et al. 2004; Matsuda et al. 2020), the relative stabilities of different gene products interact to shape clock tempo. This is demonstrated by changing ranges of protein half-life corresponding to a period of 154 min as mRNA half-life decreases. However, gene product stability alone cannot capture slowed development across increasing genome size and nuclear volume. Simply increasing protein stability and/or mRNA stability for the BM (normal diffusion) models, shown in Fig. 2A, C, and E, cannot fully recapitulate the slowed *A. mexicanum* clock (see Supplemental Material 4 for an extended discussion). The additional delays introduced by obstructed nucleoplasmic diffusion across a large nucleus, as well as transcription of longer introns, appear to be necessary to sufficiently slow the period of gene expression. Although gene product stability acts to mediate developmental tempo, our model results suggest that across increasing genome size and nuclear volume, gene product stability does not act alone to slow developmental tempo.

## Conclusion

Overall, we show that the physical and spatial delays predicted by increased intron length and nuclear size, coupled with alterations to gene product stability that ensure the products persist for long enough to fulfill their molecular function, mathematically recapitulate the slow developmental tempo found in species with large genomes. Empirical work that seeks to validate our model will focus on: (1) experimentally confirming that *hes5.7L* and *hes7* act as the segmentation clock genes in *X. laevis* and *A. mexicanum*, respectively, (2) measuring nuclear position and mRNA export time for the clock genes in both species, and (3) measuring biosynthesis rates, as well as transcript and protein stability, for the clock genes in both species. If our mathematical predictions are borne out empirically, we will conclude that the longer intracellular travel distances accompanying evolutionary increases in genome and cell size slow development by altering gene expression kinetics.

## Supplementary Material

obae021_Supplemental_File
